# Towards an Improved Understanding of the Anorexia Nervosa and Autism Spectrum Comorbidity: PEACE Pathway Implementation

**DOI:** 10.3389/fpsyt.2020.00640

**Published:** 2020-07-07

**Authors:** Kate Tchanturia, Katherine Smith, Danielle Glennon, Anna Burhouse

**Affiliations:** ^1^ Department of Psychological Medicine, Institute of Psychiatry, Psychology and Neuroscience, King's College London, London, United Kingdom; ^2^ National Eating Disorder Service, South London and Maudsley NHS Foundation Trust, London, United Kingdom; ^3^ Northumbria Healthcare NHS Foundation Trust, Patient Experience Department, Wansbeck General Hospital, Ashington, United Kingdom

**Keywords:** eating disorders and autism pathway, treatment, implementation, comorbidity, innovation, qualitative study

## Abstract

This paper describes an eating disorder service development pilot project helping clinicians implement evidence-based research for patients with anorexia nervosa and autism spectrum condition comorbidity. Currently, there are no clear guidelines or recommendations for people who have the developmental condition of autism spectrum condition and a comorbid eating disorder. The Maudsley eating disorder team is pioneering a tailored approach of adaptations for this autism and eating disorder comorbidity to improve and adapt evidenced-based treatments and improve the experience for patients, families, and clinicians involved in their care. This paper aims to support the clinical and research community to implement some of the learning and new strategies developed through the PEACE pathway. The authors aim is to collaborate with teams nationally and internationally to scale up the project to benefit patients with this comorbidity.

## Plan

### The Problem

The link between autism spectrum condition and eating disorders is increasingly researched and in recent years has been receiving more clinical attention. With estimates of comorbidity up to 37% (1), extensive research has been conducted on cognitive profiles (2), socio-emotional processing (3), clinical naturalistic studies (4), and epidemiological studies (5, 6). The research in the field has indicated that the overlap in the two disorders seems to be mostly related to high levels of cognitive rigidity, attention to detail (2, 7), sensory sensitivities (8), and poor social functioning (9–11).

Patients with the comorbidity have been found to fare worse in treatment programs, with poorer outcomes, heightened presentation, and longer in-patient admissions (4, 6). From our own internal audit data, we can see that these patients have longer stays in treatment as well and higher percentages are being seen in the more intensive in-patient settings. This evidence leads us to believe that there is a high percentage of this comorbidity seen in cases of severe and enduring eating disorders and that urgent attention is needed to support this client group, in order to improve their treatment outcomes.

This manuscript summarizes our attempt to translate research evidence into the clinical work we do with patients with comorbid eating disorders. Autism spectrum conditions (preferred term by people with lived experience) are developmental conditions where traits remain stable over lifespans. Recognizing and acknowledging these traits and their stability in eating disorder patients is the first step in educating healthcare professionals in how best to support the unique needs of those on the autism spectrum. Eating disorders, on the other hand, are serious mental health conditions with physical and psychological risks needing treatment. In the clinical pathway development for Autism Spectrum Condition (ASC) and Anorexia Nervosa (AN), we take the view that treatment as usual can be modified to support people with ASC to recover from an eating disorder.

### Needs Assessment to Address ASC and Eating Disorder Comorbidity:

Recent interviews with all major stakeholders in supporting the comorbidity effectively (patients themselves, their carers, and their multidisciplinary clinicians), which we refer to as the “stakeholder needs assessment triangle,” suggest that there is an urgent need for guidance in this treatment pathway (12–14). These interviews suggest several overlapping themes which all the groups agree on such as: acknowledging a link between the two diagnoses; the need to address neglected sensory sensitivities; that therapeutic engagement takes a longer time to build with therapists; that an individualized and flexible approach needs to be taken in treatment; that there are currently barriers for these patients, namely difficulties in gaining a diagnosis and no clear pathway being available; and finally that clinician training and support around the comorbidity is needed (12–14). In addition, thought needs to be given to ensuring reasonable adjustments are made to outpatient and inpatient treatment areas, to reduce stress levels and support treatment.

There is an urgent need to identify these patients and support their additional needs to ensure patient-centered care and optimal treatment outcomes. With no current guidelines on how to best approach the comorbidity, a novel pathway has been co-produced by patients, clinicians, carers, and researchers within South London and Maudsley NHS Trust Eating Disorders Services. This clinical pathway aims to address all the issues identified within the main stakeholder interviews in order to improve treatment outcomes of the patients with the comorbidity (12–14).

This clinical pathway has been developed using the Institute for Healthcare's Model of Improvement quality improvement methodology, using an iterative Plan, Do, Study, Act (PDSA) format to introduce change and to co-produce the work with people with lived experience and with healthcare professionals. PDSA is implemented in cycles where each development once implemented is continuously assessed and improved in a structured way on a small scale before full-scale implementation. This allows change and feasibility to be assessed in the least disruptive way. We believe this will help define the model and assist with scaling up this program to other eating disorder treatment sites. This article has been structured into the “Plan, Do, Study, Act” template, although retrospectively this information does not slot as easily under the headings as our “Do”-ing and “Study”-ing were taking place alongside each other.

We called the project the project Pathway for Eating disorders and Autism developed from Clinical Experience (PEACE) pathway. This name was chosen by a patient vote as being the most popular of a series of abbreviations. This paper outlines how we have implemented PEACE pathway with the aim of improving care for patients with the ED and ASC comorbidity. We further want to demonstrate that by following our steps and strategies from our experience other clinical teams can “give PEACE a chance”.

## Do

### Clinician Training

As identified in the triangulated stakeholder's interviews, there is a need for clinicians additional training for all members of the multidisciplinary team, and better confidence to manage the ASC and ED comorbidity. This training would not only address this theme, but also others highlighted in the triangulated stakeholder's assessments such as: acknowledging the link, acknowledging atypical sensory profiles, aiding with therapeutic engagement, suggesting ways of working in an individualized and flexible way with each individual. Research suggests that there is a lack of recognition and understanding around the comorbidity and how the two diagnoses interact (15). We have therefore introduced a regular training program for clinicians in South London and Maudsley NHS foundation specialist eating disorder service including inpatient, outpatient, and daycare programs. This training series aims to translate information provided by experts and clinicians in the autism field into applicable adaptations for clinical care on eating disorder wards. Evaluations were taken of each training session providing both qualitative and quantitative feedback. We started with the tools of Assessment of Autism including training in ADOS-2 (Autism Diagnostic Observation Schedule-2) and ADI-R (Autism Diagnostic Interview-Revised). Feedback for these sessions suggested that clinicians gained valuable experience from these two training blocks, most of them commented that interpretation and a good understanding of ASC clinical assessment gave them better insight and understanding of the ASC presentation. Clinical assessment sessions were found most informative in the area of female ASC, where clinicians learned to better understand how females with autism often camouflage their autism symptoms. Furthermore, feedback suggested that the questions such as “how do you differentiate between a friend and colleague” and “How do you feel when you are (insert emotion)” used in the assessments, particularly the ADOS-2, invited clinicians to think about how to approach certain topics essential to therapy, such as relationships and emotions, and gave a better insight into behaviors of those patients with comorbid autism. Later training sessions focused on adaptations applicable for clinicians. These included various therapeutic modalities and how current clinicians within the autism field adapt to particular patients (CBT, DBT, formulation-based approach, CRT, and CREST training). Further training included learning about specific sensory adaptations for patients and how these would be dealt with in sessions and potential environmental adaptations from experienced occupational therapists working in the ASC field. The knowledge gained from these sessions has allowed our multidisciplinary health professional team of clinicians to build up their “toolbox” of techniques that may help and support individuals, tailoring treatments to the individual and being flexible in their approach as highlighted as a necessity in the triangulated stakeholder interviews.

**Figure 1:**
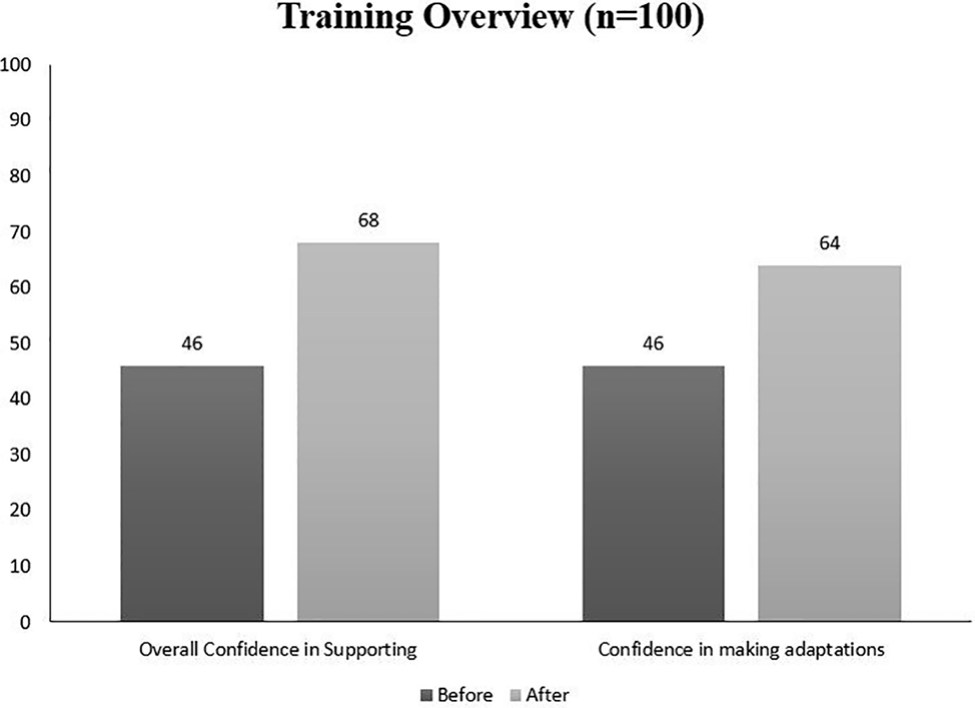
Training feedback graphs.

Clinician confidence and skilling the team up in terms of flexibility and useful adaptations were our main drivers when introducing our PEACE training sessions. Feedback from 100 attendees of these sessions suggests that clinicians' overall confidence in supporting the comorbidity increased from an average of 46/100 to 68/100 (**Figure 1**). In terms of confidence in making adaptations in treatment for patients with the comorbidity, there was a similar increase in confidence from 45/100 to 64/100 (**Figure 1**). Overall, 92% of clinicians agreed that the training sessions equipped them with knowledge, skills, and tools to support patients with the comorbidity and 97% would agree that these training sessions should be recommended to other eating disorder clinicians. The highest confidence changes were after our Formulation adaptation training which saw an average of 35-point increase per person. In addition, the clinical team provided qualitative feedback on the training sessions (e.g. “thanks for recommendation to use more drawing,” “I will remember now, less words more visuals,” “I will pay more attention to sensory difficulties,” “brief sensory assessments will be helpful in my work”).

### Clinician Support

As well as introducing autism-specific training, we have also implemented weekly team huddles or “snapshots” which last for 15 min. There is a weekly huddle on the inpatients and step-up unit and one for outpatients and the daycare service. These huddles aim to address many of the themes identified in the triangulated stakeholders needs assessments such as acknowledging the link, offering a clear pathway, supporting clinicians and as a result improving outcomes of treatment adaptations. The purpose of these huddles is to provide support, keep continuity, assess additional needs for the treatment team to improve the quality of the care for the ASC/ED comorbidity. This can be through informal groups supervision, where cases are briefly discussed or formulated as a team. We also think about developments of the PEACE Pathway within these 15 min, either what changes are new or how we can improve current care and treatment plans. The materials we have developed, such as rapport enabling psychoeducation will be discussed and edited by the team in the huddles, ensuring all knowledge developed from clinical experience is collated. Many of these materials along with others are now freely available on our PEACE website. In addition, once a month at each site, we run an extended huddle of one hour where a case study is presented. This will have input from all members of the multidisciplinary team. We observed there were many benefits from these extended huddles for the team including better communication between multiple disciplines involved in the care of the patients (medical doctors, nurses, psychologists, occupational therapists, nurse assistants, admin, and housekeeping staff were also increasingly involved in the huddles particularly with environment adaptations discussed later). As well as this, we found the huddles a useful place for staff to reduce their own stimulus overload, in order for them to be able to do the same for patients.

### Patient Identification

Eating disorders are predominantly seen as “female” disorders. In contrast, autism is often seen as a “male” condition. This inevitably creates a bias in the identification of female eating disorder patients with autistic traits, let alone barriers created due to “diagnostic overshadowing” (16). This is where, in our case, the eating disorder and its symptoms would overshadow the autism, often leading to a missed diagnosis. All three stakeholders suggested how difficult it currently is to be identified as autistic and how difficult it is to peruse a diagnosis. Furthermore, clinicians found there was no clear pathway for patients who were identified. As part of our pathway, we have introduced a basic screening measure for all patients starting treatment across our Eating Disorder treatment programs. The AQ10 (Autism Quotient-10) is a ten-question questionnaire which is a shortened version (17) of the AQ50. This measure is not a diagnostic tool but has been found to be an accurate measure in predicting the likelihood of autism. An additional “screener” we used, was clinician's instinct, we updated our admission assessment to highlight the comorbidity to the assessing clinician. Research has shown that clinicians are often very good predictors of autism (18). This instance of high clinician predication came about after the implementation of the pathway, suggesting that initial “instinct” may be lacking and that after training and open discussions around assessments, clinicians may feel more equipped to recognize autistic people. After the screener, and when potential patients were flagged up, they would often undergo an ADOS-2 assessment. When scoring the ADOS-2, we would always use the updated algorithm (19) as it has been found to have increased sensitivity for females with anorexia (20). This report gave the team a more detailed sense of where this particular individual could be supported, be it in relationships, emotion regulations, or social aspects of daily functioning. For example, if it came to the assessor's attention that the individual particularly struggles with identifying emotions, this would inform clinicians that Cognitive Remediation and Social Skills Training (CREST—more detail in *Psychological Treatments* section) would potentially be a suitable intervention. A further identification tool used was a sensory sensitivity scale which was given to all patients at the start of their treatment. This was a useful tool as the ADOS was not designed to be sensitive to populations who have sensory impairments (21), as sensory atypicality was only recognized formally as being a trait of those with autism in since the assessment was created (22).

### Addressing Neglected Sensory Sensitivities

The need to attend to atypical sensory profiles of this patient group was agreed by all parties (patients, clinicians, and families) in our qualitative research studies (12–14). This is of little surprise, as research has shown that treatment settings are often over-stimulating for patients on the autistic spectrum (23). To address this, we worked closely with the National Autism clinical program in our NHS Trust and had consultations with invited experts from the National Autistic Society. We wished to adapt our ward environments to tailor to the sensory needs of the patients with the comorbidity, whilst also balancing the needs of the patients who do not have atypical sensory profiles. Using the co-production nature of our design this was achievable with regular feedback and decisions being made by all patient groups as a whole. For example, all neutral and low stimulating colors were identified for the new color scheme of the ward. All patients then had a say on which neutral color scheme we would select to make the decision inclusive. When implementing the change, one useful thing we found on our inpatient ward was to do this over a weekend. This way, many of the patients were on home leave and there was minimal disruption to the daily routine, especially when refurbishing the dining room. Other necessary environmental adaptations included providing all staff with key-covers for noise reductions, de-cluttering our ward spaces, and “going easy on the walls” (parent quote in [14]).

The sensory screening tool which we developed has been implemented as a pragmatic short assessment to explore over and under-sensitivity in the domains of smell, touch, taste, sight, and sound (24). This tool has been piloted by our patient group over the past year and preliminary findings suggest that 37% of our inpatient clinical group have possible ASC comorbidity. The majority of those with the comorbidity have self-reported problems with touch, and textures when compared to other inpatients without the comorbidity. However, in the domain of taste sensitivity, those with the comorbidity seem to have lower on average taste sensitivity than their other inpatient counterparts. There is also room for expansion on the screener and many patients with ASC have highlighted the preference for bland, soft-textured meals. This information is valuable, especially when taking food choices into account.

In light of sensory information, we have developed a specialized PEACE menu. This was co-produced by dieticians and patients to create a menu that addressed common sensory complexities in patients, whilst also ensuring all nutritional needs were met to ensure proper nourishment of this patient group. Meals on the menu were commonly bland, soft textured and as simple/predictable as possible. The predictability was ensured by having menu items which would not vary much such as mashed potatoes or a cheese sandwich. An example of pathway development within our huddle was just before the implementation of the menu, we brought it to the huddle for discussion and feedback. One of our nursing colleagues suggested we add photographs of each meal as it would be presented to decrease as much anxiety as possible and to increase predictability. This idea was then co-developed by patients who were using the PEACE menu. Sensory sensitivities around food are important to be aware of when supporting autistic people in their eating disorder recovery. We have received positive feedback from our dietetic team about how the PEACE pathway has aided their clinical practice: “As a dietitian, involvement with PEACE increases my understanding of how people with an eating disorder and ASD experience food and eating, and this helps me to better support recovery from the eating disorder.”

Further adaptations implemented based on our sensory findings included introducing a sensory box to our ward environments. These boxes include several items requested by the patients with the comorbidities, and as they are often costly items, we suggest it is used as a “try before you buy” box. Items in our box included weighted blankets, ear defenders, and essential oils. Patients are also encouraged to support their own sensory needs in Occupational Therapy by creating their own Sensory “First Aid” Kits. The idea behind these is that in times where sensory stimulation is too much, or not enough, the patient is able to ground themselves on their own. In these boxes, patients are encouraged to collect things that will stimulate different senses, for example dried lavender, some putty or bubbles, etc.

Our Occupational Therapists have also developed a Sensory Group. This group is run over the course of 5 weeks and each week addresses a different sense. The aim of each session is to provide psychoeducation around each sense, allow patients to explore their sensitivity and preference to each sense in a safe, explorative way.

The psychology team further developed a one-off sensory workshop using psychoeducation and experiential content across different eating disorder treatment programs (inpatient, daycare). This workshop is inclusive of all patients, with or without the comorbidity, and is aimed at increasing each participant's knowledge and understanding of their own sensory needs and sensitivities. Using this information, participants are then encouraged to think of the self-soothing nature of meeting these sensory needs and experiential ways of addressing these are suggested and explored together.

### Psychological Treatments:

Based on our naturalistic studies we evaluated how patients with AN respond to adjunct therapies based on remediation principles of Cognitive Remediation Therapy (CRT) and Cognitive Remediation and Social Skills Training (CREST) with or without ASC features. In general, we engage patients immediately after admission (regardless of BMI) in psychoeducation and experiential exercise-based cognitive and emotional training programs in individual and group formats. Based on our preliminary findings people with comorbid ASC and AN find these therapies helpful, however self-report and neuropsychological outcomes after therapy significantly change only for individual CRT and CREST. Evaluation of the group versions demonstrated that although there were no statistically significant changes in the self-report questionnaires before and after the group, patients' qualitative feedback was seemingly positive (25–27). Interestingly, qualitative feedback from the patients with high ASC traits suggested that they found CRT and CREST groups safe, easy to attend, and not threatening, suggesting that it might be a helpful setting to begin to tolerate social situations.

Feedback from our clinical team suggests that using classic Motivational Interview techniques for these patients is often extremely challenging, which was reflected in the desire for training and more research in this area is needed. One of the possible explanations could be that motivational interviewing can use open-ended questions, metaphors, choices which is hard to access for people with ASC features due to preference to concrete, specific, simple, and literal language use.

As well as identifying which therapeutic interventions have been successful with this patient group, we have found various individualized strategies can be successful therapeutically (28). Our clinicians have reported that being flexible in various strategies such as the length, pace, and focus of sessions can have a significant impact on outcome and experience for the patient and therapist. For example, someone may have a particularly slow processing speed, who may need slightly longer or more session. On the other hand, someone may have a very short concentration span and may need to have short breaks throughout the session.

In the PEACE pathway we have developed psychoeducation materials for patients, as well as supporting adapted materials. We have adapted and developed an autism-friendly welcome pack for our patients. These are to be sent out pre-admission to make a highly anxiety-provoking situation less anxiety-provoking. This pack includes all information patients being admitted to the ward might need, including what an average bedroom looks like, what an average weekly schedule would be, when they might need to fill their own time and suggestions on what to do in this time, a site map and other information. Communication passports have also been developed for each patient. These communication passports are collaboratively completed so that personal information to do with communication styles, preferences, and goals, are fully understood and accessible to all clinicians involved in the patient's care.

### Carers' Support:

The third stakeholder from our stakeholder needs assessment triangle: the carers who have loved ones with the comorbidity. Implementation of carer support is in its infancy, and it is a direction we wish to explore more. We deliver monthly Carers' workshops in our department across the eating disorder program. The purpose of these is to support carers of our patients (e.g. how the brain works when people are malnourished, how to communicate effectively, how to support eating). Our carers' workshop now includes information on the comorbidity, to raise awareness and understanding of carers who attend. Furthermore, the information provided at these workshops and handouts have been made user-friendly, as autism is known to have a heritable aspect and to be considerate to carers who may also have autistic features. Other adaptations to our carers' workshops include the addition of a new “animal” metaphor/s which are used to represent different carers' styles (see (29) for original animal metaphors). In referrals of family therapy across the service (for example, from inpatient to day-care), referrals now explicitly state if a patient has an autism diagnosis or if they or their family members display high ASC. As well as this, clinicians detail any particular adaptations that have been helpful to this patient and their carer/s. Adaptions may include seating positions, lighting, regular appointment times, written information and debriefs, and communication passports. In light of the global pandemic, our PEACE team have also offered virtual coffee mornings to carers to meet and discuss, facilitated by a psychotherapist. Due to its popularity and success, we hope to develop this idea into a more permanent fixture.

### Resources for All Stakeholders:

We understand that the stakeholders of the pathway are not only those involved in the South London and Maudsley NHS Foundation Trust, and as a result we have developed materials and resources to share with others based on the learnings of our PDSA “cycle” implementations on what resources where needed for which group of stakeholders. We have a PEACE book currently in publication as well as a newly launched website (www.PEACEpathway.org) which has materials for all three groups of stakeholders freely available. We are hoping to develop this in the future to include a forum to increase our PEACE community-feel.

## Learning Points, Future Directions, and Dissemination

### Study

Through implementing this pathway, we have learned a lot about the dynamics and individual contextual challenges of eating disorder clinical service environments at the Maudsley hospital. As part of this development, staff and patients have faced and overcome adversities and as a result learned several important lessons. One thing we found challenging, was that there was some staff resistance. This was mainly due to an increasing perception of workload or “another level” added to their role. In order to successfully implement the changes, a network was established and continues to grow so that the pathway can continue to develop, adapt, and change after the pilot study with its external support. The huddle being open to all staff and sharing case studies went a long way in obtaining staff buy-in of the need for the pathway. This was done with regular exposure to these patients and their complex cases through case presentations. Discussing these cases as a multidisciplinary team and having all voices heard and valued was essential. Appointing champions (mini project leads) was another important step. Having various champions such as a “sensory champion” and a “dining room champion” has given a sense of belonging and responsibility, without too much-added burden as the load has been spread. We have also increased our forms of communication, to make sure we reach everyone, even those too busy to attend the huddles or those not on the right shift pattern. We have done this through extensive communications amongst busy clinical teams: monthly newsletters, huddles minutes, posters, and ways to recognize us such as pens and badges setting up PEACE twitter. These forms of communication make sure that everyone knows their thoughts and opinions of the pathway are valued and that even if they are not in the meetings they still belong in the pathway. Through these techniques, we have generated interest in our pathway and more staff are becoming actively involved. This has implications for further spread of the model, as each adopting site will have its own individual contextual factors to navigate (30). Each adopting team will need to use the tools developed by the PEACE pathway to develop their local solutions, highlighting the importance of having a core group of interested staff or champions willing to engage in coproduction with patients to make positive reasonable adjustments to service provision and environments.

In terms of patient resistance, this has mainly been from those without the comorbidity. As mentioned previously, although most adaptations are tailored to those with the comorbidity, they benefit all patient groups. Examples of this include the sensory tool kits, where grounding techniques can benefit all patients. The PEACE menu is also available to all patients who are not on the spectrum three times a week. This allows those who may be particularly anxious about a meal or are in need of something predictable, an opportunity to do have this. Involving all patients in decisions has also been very important in ensuring all voices are heard. Regular feedback is also asked for from all patients and focus groups to create meal-support materials included for all patients.

### Act

The details of implementation currently focus mainly on two parts of the needs assessment of our original triangle: patients and clinicians. In future adaptation, we will further develop a protocol for carers support. We have outlined above the implementations currently taking place for carers, however, we would like to support this group of carers more. We aim to do that by developing a platform to support and skill up carers. As well as supporting carers, we also hope to look at the comorbidity in young people and to be able to adapt the PEACE pathway for children and adolescents who are also struggling with a lack of individualized and flexible care. We also note that there is emerging evidence for other forms of Eating Disorders such as bulimia nervosa and binge eating disorders being equally comorbid with autism as anorexia nervosa. We would aim for future directions of the pathway to ensure all eating disorder groups are benefited and included.

### Dissemination

We hope that with this information other eating disorder services will be able to “give PEACE a chance” and take forward our implementation strategies and improve them, through further testing and iteration of the model. By using a quality improvement approach of implementation, we hope to learn if there are “core” elements to this process that all sites use and map the local adaptations. There is no “correct” way to support this comorbidity group and each implementation step is a learning process which we hope other services will be part of. With new ideas and techniques being introduced we hope to build a supportive, inclusive community.

## Data Availability Statement

The datasets generated for this study are available on request to the corresponding author.

## Ethics Statement

The studies involving human participants were reviewed and approved by South London and Maudsley NHS Trust. The patients/participants provided their written informed consent to participate in this study.

## Author Contributions

KT is a principal investigator of the PEACE pathway project. KS is a project manager collecting the data and managing day-to-day activities in the project. AB edited and provided coaching for the project implementation. KT developed the study protocol, supervised team. KT and KS wrote the paper. All authors contributed to the article and approved the submitted version.

## Funding

The Health foundation an independent charity committed to bring better health care for people in the UK (Ref : AIMS ID): 1115447 and the Maudsley Charity is an independent NHS mental health charity which works in partnership with patients and families, clinical care teams, and researchers at South London and Maudsley NHS Foundation Trust, the Institute of Psychiatry, Psychology and Neuroscience, King's College London, and community organizations, with a common goal of improving mental health, to support innovation, research and service improvement.

## Conflict of Interest

The authors declare that the research was conducted in the absence of any commercial or financial relationships that could be construed as a potential conflict of interest.
